# Valorization of cigarette butts for synthesis of levulinic acid as top value-added chemicals

**DOI:** 10.1038/s41598-021-95361-4

**Published:** 2021-08-04

**Authors:** Amelita G. Laurenza, Onofrio Losito, Michele Casiello, Caterina Fusco, Angelo Nacci, Vincenzo Pantone, Lucia D’Accolti

**Affiliations:** 1 Ambra SRL, Via Mitilini 11 Pomarico, 75016 Matera, Italy; 2 Dipartimento di Chimica, Università degli Studi di Bari “A. Moro”, Via Orabona 4, 70126 Bari, Italy; 3 Bari Section, CNR-Istituto di Chimica dei Composti Organometallici (ICCOM), Via Orabona 4, 70126 Bari, Italy

**Keywords:** Environmental sciences, Chemistry

## Abstract

Unprecedented in the literature, levulinic acid (LA), one of the top value-added intermediates of chemical industry, is obtained from cigarette butts as cellulose feedstock by means of a one-pot hydrothermal process carried out at 200 °C for 2 h and catalysed by phosphoric acid. The protocol avoids the use of more aggressive and toxic H_2_SO_4_ and HCl, that are generally employed on several cellulose sources (e.g. sludge paper), thus minimizing corrosion phenomena of plants. Neither chemical pre-treatment of butts nor specific purification procedure of LA are required. Notably, by simply modifying acid catalyst (e.g. using CH_3_COOH), another top value-added fine chemical such as 5-hydroxymethylfuraldehyde (HMF) is obtained, thus widening the scope of the method. Being cigarette filters a waste available in quantities of megatonnes per year, they represent an unlimited at no cost source of cellulose, thus enabling the up-scale to an industrial level of LA production.

## Introduction

The conscious use of the planet's natural resources has become mandatory to ensure the survival of life on earth. To this end, the imperative contribution that the scientific community can provide is to develop new sustainable process and materials, with a significant impact on the social level and with a reduced environmental repercussion. In this context, the tuning of new chemical approaches to exploit the waste has an extreme importance in the realization of the virtuous path that Circular Economy is encouraging, to create environmental and social benefits, in a “Rethinking Progress” approach for sustainable development and sustainability.

Exploiting biomass is the true weapon to face this challenge, the true road for producing energy, fine chemicals and bio-based manufacts in a sustainable manner, thus definitively eliminating the dependence on fossil sources, without loss of soil^1^.

Important examples of biomass are wood and energy crops, such as for example soy, useful for producing biofuels but also bio-based chemicals and polymers^2^. Recent years have witnessed a rapid growth in the production of fuels^3^ and materials entirely deriving from biomasses^4^. However, this has led to many troubles such as improper exploitation of soils (non-food applications), increase of raw materials price (especially in Third World countries), biodiversity reduction, soil erosion and increased risk of insects and bacteria that destroy crops^1^.

Biomass wastes can be the right solution to these problems^2^, constituting a widely available and no cost reservoir of carbohydrates, lipids and proteins, with possible on-site processing, coming from scraps of forests, yards, farms, or municipal waste foods, the proportion of which has been estimated at hundreds of megatonnes (Mt) per year around the world^5,6^.

**Figure 1:**
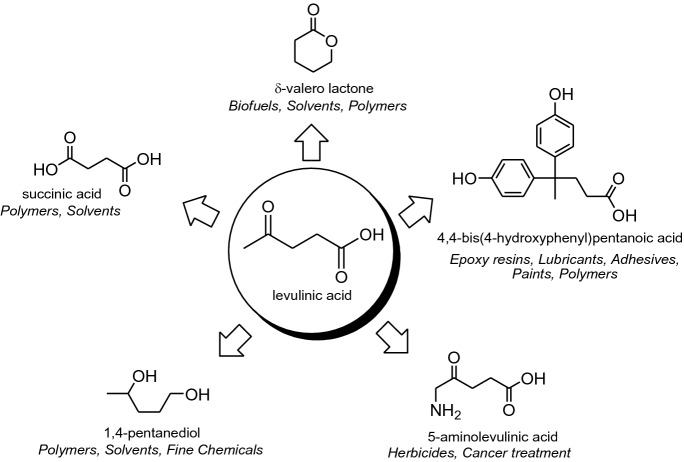
Levulinic acid as a key industrial intermediate.

Carbohydrates, the main component of these vast reservoir, can be converted via biological or chemical routes into Levulinic Acid (LA)^7,8^, which is considered one of the twelve most promising industrial bio-intermediates and amongst the most innovative building blocks of chemical industry, due to its conversion in several high-value bio-based chemicals and materials (Fig. 1)^7^. The main end users of LA are agricultural, pharmaceutical, and cosmetic sectors, although this natural molecule also contributes to the creation of new ecological fuels, fertilizers, and pesticides. It is also used in the biodegradable plastics field and as intermediate element for creating high-performance plastic materials, medicines, and many other new concept "green" products, thus allowing to broaden its scope of application. According to the most recent studies, it is estimated that the world market demand for LA will grow 150–200 times over the next 7–8 years^8^.

**Figure 2:**
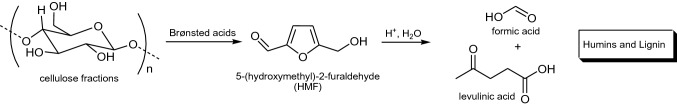
Two-step synthesis of Levulinic Acid.

The well-known approach to convert lignocellulosic materials (wood, paper, food crops wastes) into levulinic acid is the thermal treatment with strong Brønsted acids (e.g. H_2_SO_4_) as homogeneous catalysts^9,10^. To date, a two-step continuous process is used to obviate the deterioration of the plants. Hemicellulose and cellulose fractions of biomass are preliminarily hydrolyzed in a first reactor (at 210–230 °C, for few seconds in the presence of 1–5% of mineral acid) producing hydroxymethylfurfural (HMF), that is removed in flow feeding continuously a second reactor where it is further hydrolyzed to produce LA (Fig. 2)^10–16^.

Despite the high yields, this strategy is difficult to apply at an industrial level, due to the harsh conditions and plants corrosion^10^. To date, only few companies can produce LA at commercial scale directly from biomass^10,17^. In recent years, much attention has been paid to producing LA by means of milder and more eco-sustainable conditions^16^, for example employing heterogeneous acid catalysts and green solvents such as water or ionic liquids (ILs)^8,17^.

As regards the cellulosic starting material, beside agricultural scraps^10^, municipal paper wastes are gaining attention^17^. Among these latters, cigarette filters represent a neglected and no cost reservoir of cellulose acetate^18^, that is virtually boundless if considering that about 5.5 trillion cigarettes are produced each year^19–21^. Notably, used cigarette butts (CBs) are considered a dangerous waste, because of the content of organic and heavy metals contaminants, therefore their use as starting raw materials is rather complex and essentially limited to production of asphalts, mesoporous carbon, and cellulose pulp^16–22^. Recently, bioethanol has been produced by fermentation of cellulose obtained by deacetylation of CBs^22^, but no attempts have been reported until now on their use to produce LA or other fine chemicals.

Following our ongoing interest in developing green protocols obeying to circular economy principles^23,24^ we report herein, unprecedented in the literature, a protocol that exploits cigarettes filters as source of Levulinic acid avoiding the strong acidic conditions and extendable at an industrial level.

## Results and discussion

In the proposed procedure the preliminary digestion with strong acids (H_2_SO_4_) was circumvented using a one-pot procedure involving H_3_PO_4_. Notably, being composed by cellulose acetate, CBs fibres must be deacetylated before undergoing deconstruction (swelling of cellulose chains) and hydrolysis. On these bases, phosphoric acid appeared the suitable choice as it is particularly efficient in changing the structure of cellulose by breaking interchain hydrogen bonds favouring deconstruction^13^. In addition, among the widely available mineral acids, H_3_PO_4_ is strong enough to give efficiently deacetylation, deconstruction, and hydrolysis, but is simultaneously low toxic and much less corrosive than HCl and H_2_SO_4_^25^, which is also known, the latter, to give partial carbonization of organic substrates.

**Figure 3:**
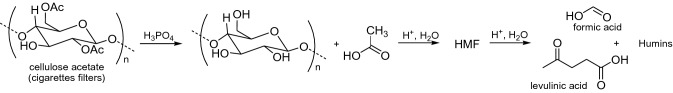
One-step synthesis of Levulinic Acid.

Preliminarily, fresh and used filters were subjected to characterization by LCSM technique for determining fibres morphology, and by ICP/MS and COD analyses for evaluating amounts of contaminants (see supplemental information). According to most of reported procedures, catalytic hydrolysis experiments were conducted in a batch reactor processing 250 mg of filters in 15 mL of aqueous H_3_PO_4_ at different times and temperatures^14^ (Table 1). Formation of Levulinic Acid was surveyed by GC/MS and NMR techniques. Both unsmoked and smoked cigarette butts were tested as source of cellulose biomass, whereas acetic and formic acids were formed as by-products together with HMF as an intermediate (Fig. 3)^16,26^.

**Table 1:** Synthesis of levulinic acid from cigarette filters.

Entry	Filters weight (mg)	Catalyst (M)^b^	T (°C)	Time (h)	Levulinic acid yields	
					(%)^c^	(% filter wt)^d^
1	251.24	–	200	2	–	–
2	253.38	H_3_PO_4_ (1.5)	160	2	–	–
3	250.38	H_3_PO_4_ (1.5)	180	2	22.5	10.43
4	252.85	H_3_PO_4_ (1.5)	190	2	24.7	11.42
**5**	**253.19**	**H**_**3**_**PO**_**4**_** (1.5)**	**200**	**2**	**36.7**	**17.32**^**e**^
**6**	**250.57**	**H**_**3**_**PO**_**4**_** (1.5)**	**240**	**2**	**43.9**	**20.28**^**e**^
7	252.93	H_3_PO_4_ (1.5)	260	2	35.4	16.36^f^
8	101.70	H_3_PO_4_ (1.5)	200	2	24.3	11.28
9	200.80	H_3_PO_4_ (1.5)	200	2	35.7	16.47
10	299.50	H_3_PO_4_ (1.5)	200	2	30.1	13.90
11	251.55	H_3_PO_4_ (0.7)	200	2	25.2	11.46
**12**	**250.96**	**H**_**3**_**PO**_**4**_** (2.0)**	**200**	**2**	**41.4**	**19.15**
13	250.37	H_3_PO_4_ (1.5)	200	1	17.3	8.00
**14**	**251.04**	**H**_**3**_**PO**_**4**_** (1.5)**	**200**	**6**	**49.6**	**22.94**
15	252.52	CH_3_COOH (4)	200	2	–	–^g^
16	254.06	H_2_SO_4_	200	2	63.1	31.6

^a^Procedure as reported in experimental section.

^b^Volume = 15 mL.

^c^Referred to theoretical amount of LA (see “Materials and methods” section).

^d^Referred to the filter weight^14^. All yield values were obtained based on three replicate experiments (SD ± 2.0).

^e^Humines = 79 mg (32% w/w). Humines = 173 mg (69% w/w).

^f^Humines = 205 mg (82% w/w).

^g^5-hydroxymethylfuraldehyde (HMF) was the main product (see MS spectrum in Supplemental Information).

Blank reaction carried out in the absence of H_3_PO_4_ led to the complete recovery of unreacted filters, thus confirming that Brønsted acids are true catalysts for the process (Table 1, entry 1). The successive experiments, aimed at evaluating the temperature effect, showed that reaction requires a minimum heating at 180 °C, displaying the maximum yield of 43.9% in levulinic acid at 240 °C (Table 1, entries 2–6). However, the increase of temperature led also to significant increments of humins by-products (Table 1, entries 5–7).

Amount of starting material proved to be a further parameter affecting reaction yield, with 250 mg representing the optimal value (Table 1, entries 5, 8–10). Catalyst loading and reaction time were also investigated. In the former case, the increment of concentration of aqueous H_3_PO_4_ up to 2.0 M resulted in a neglectable increase of yield in levulinic acid respect to preliminary experiments, thus suggesting that 1.5 M is the best value (Table 1, entries 5, 11–12). In contrast, much prolonged times afforded beneficial effects on reaction yields, allowing to reach a 49.6% of yield in levulinic acid after 6 h (Table 1, entries 13–14). As expected, no conversion in LA was observed when H_3_PO_4_ was replaced by a weak acid such as CH_3_COOH. Notably, in this case another value-added fine chemical, namely 5-hydroxymethylfuraldehyde (HMF), was observed as unique product, widening the scope of this method (Table 1, entry 15). Finally, reaction with sulfuric acid (entry 16 Table 1) gave 32% of yield, this result confirms that sulphuric acid can catalyse the one-pot conversion into LA with good efficiency, but the concentration of 1.5 M requested is prohibitive for an extension at industrial level, due to corrosion problems, and the two-stage strategy is a mandatory choice.

Attempts were done to increase the yields of levulinic acid, re-submitting residual humins by-products to the hydrolysis conditions at higher temperatures and prolonged reaction times. The total absence of products indicated that such conditions are not strong enough to give the cleavage of the furan-based polymeric skeleton of humins (Eq. 1).
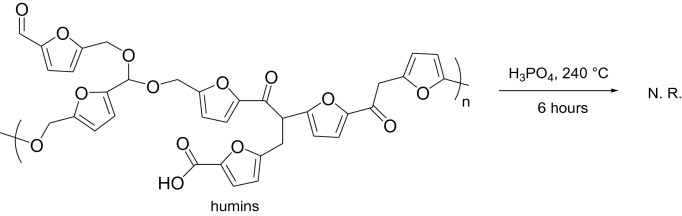


Results in Table 1 showed that the best hydrolysis conditions are 240 °C for 2 h or 200 °C for 6 h (Table 1, entries 6 and 14). Nevertheless, in both these cases, greater quantities of solid residue were observed probably due to the higher temperature and the longer times. Therefore, milder conditions of 200 °C for 2 h were selected for the successive experiments aimed at studying both real waste samples such as the smoked filters and the influence of their pre-treatment (e.g. washing).

At this end, smoked cigarette filters were washed with 100 ml of water at 80 °C for three times. The collected water fractions were extracted with ethyl acetate and the organic phase was analysed by GC/MS revealing triacetin (triacetylglycerin) as the main product, which is a humectant additive, and trace amounts of phenolic compounds.

As reported in Table 2 (entries 1–2), almost identical results in terms of yields were obtained with washed and unwashed cigarette butts.

**Table 2:** Synthesis of levulinic acid from smoked cigarette filters.

entry	Raw material (weight)	Catalyst (M)^a^	T (°C)	Time (h)	LA yields		Ref
					(%)^b^	(% filter wt)^c^	
**1**	**Washed filter (251.15 mg)**	**H**_**3**_**PO**_**4**_** (1.5)**	**200**	**2**	**33**	**15.30**	**This work**
**2**	**Unwashed filter (251.29 mg)**	**H**_**3**_**PO**_**4**_** (1.5)**	**200**	**2**	**31**	**14.31**	**This work**
3	Filter-paper cellulose^d^	H_3_PO_4_ (0.15)	200	2	–	40	^13^
4	Paper sludge	H_2_SO_4_ (0.47)	200	1	26.5	15.1	^14^
5	Paper sludge	HCl (0.47)	200	1	55.1	31.4	^14^

Procedure as reported in experimental section.

^a^Volume = 15 mL.

^b^Referred to theoretical amount of LA (see “Materials and methods” section).

^c^Referred to the filter weight^14^. All yield values were obtained based on three replicate experiments (SD ± 2.0).

^d^The pretreatment temperature and time were set to 50 °C and 24 h.

Data in Table 2 (entries 4–5) also show that this protocol can favourably compete with analogous ones reported in the literature based on the use of H_2_SO_4_ and HCl, in that very similar yields of levulinic acid can be obtained with a less corrosive acid and low toxic H_3_PO_4_ thus minimizing the corrosion phenomena^12,13,25^.

In addition, we have also avoided the thermal pre-treatment, which although it increases the yield of levulinic acid, requires a greater expenditure of energy. (entries 3).

**Figure 4:**
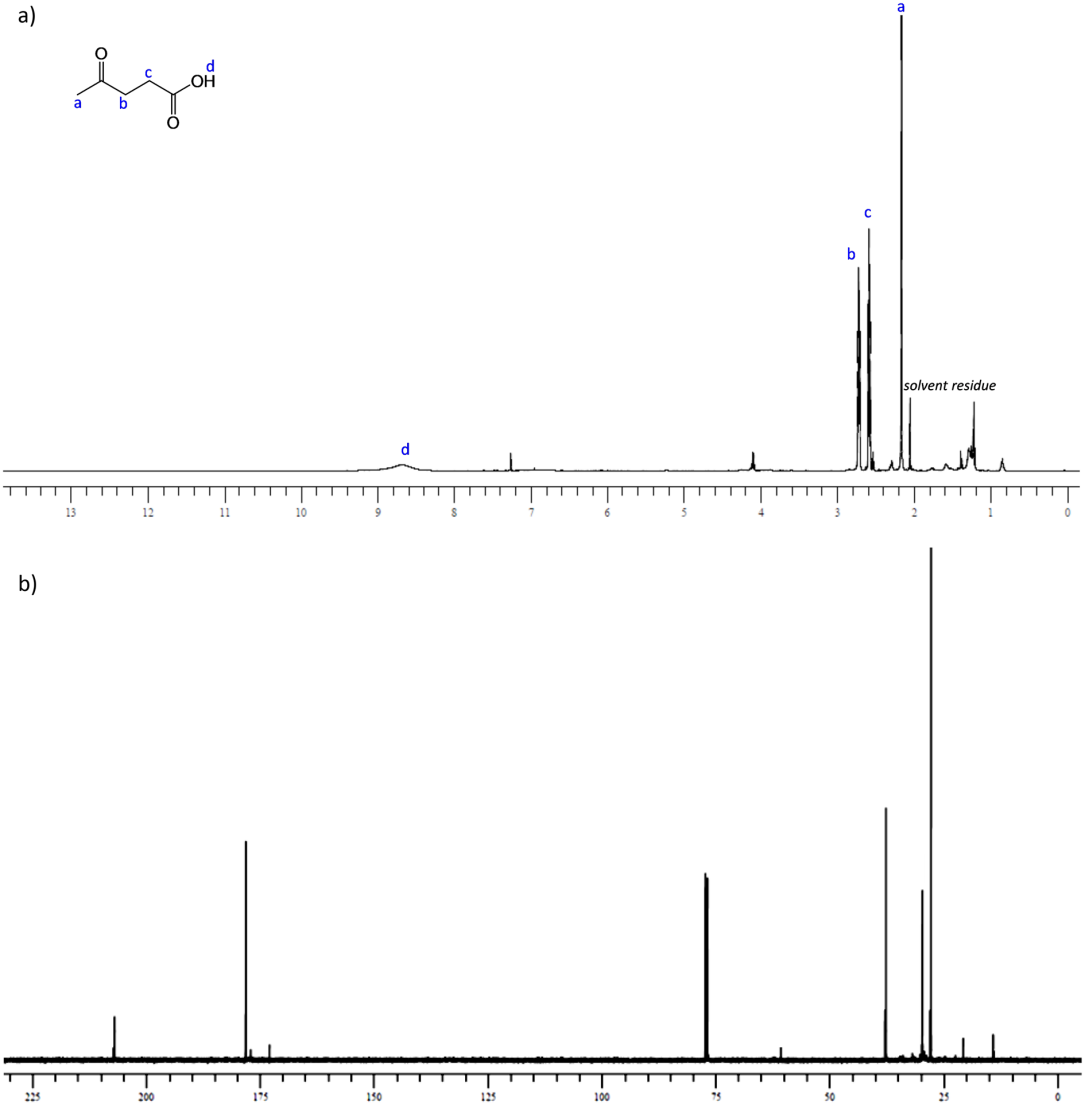
**(a)** ^1^H-NMR and **(b)** ^13^C-NMR spectra (CDCl_3_) of crude product from smoked unwashed filter.

Moreover, NMR analyses (Fig. 4) of crude reaction product of unwashed cigarette butts, revealed that levulinic acid was obtained with the same high degree of purity of that obtained with unsmoked filters (besides a little solvent residue removable in vacuo).

These results suggest that that dirt or contaminants of the smoked cigarettes do not interfere with the reaction outcome ^20,21^ and that the method is highly selective and does not require neither special pre-treatment of the starting waste material nor specific purification procedure of the reaction product.

Further aspects that represent crucial advantages for a plausible industrial application of this method concern: (i) the possibility of recycling humins wastes through thermal valorisation (burning) or syn-gas production^27^, although more recently they have been used for producing macroporous foam-like materials^28^; (ii) the prompt recycle (by distillation) of Ethyl Acetate used for extracting Levulinic acid; (iii) the possibility of recovering water phase by eliminating phosphoric acid and metal through precipitation^29,30^ and COD by Fenton treatments^31^.

These latter are two typical and cheap treatments of industrial wastewater, after which the liquid can be poured into rivers and is still considered surface water. Phosphate anions and metals are precipitated with calcium hydroxide in the form of hydroxyapatite, which is disposed of after flocculation as sludge. At the same time, most of metal contaminants are precipitated as oxides. Then, water is subjected to Fenton process (with H_2_O_2_/Fe salts) for the oxidation of the organic residues allowing the achievement of the legal limits of COD < 160 mg/L.

**Figure 5:**
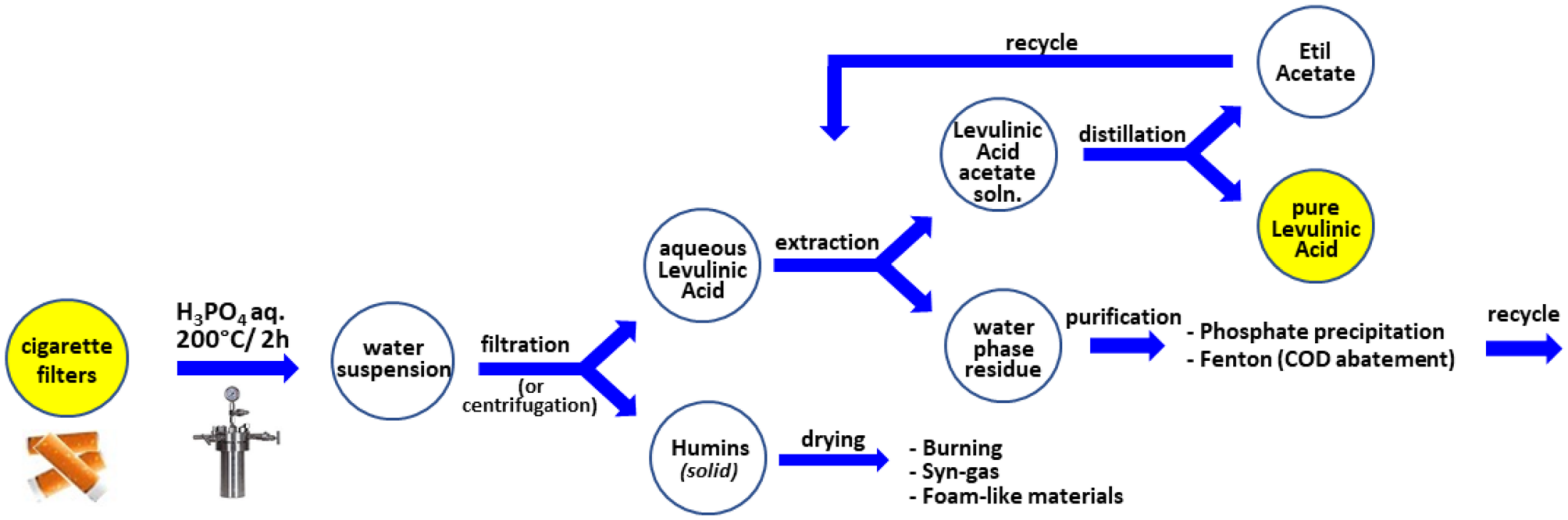
Process diagram of proposed method.

A complete process diagram of this protocol is listed in Fig. 5. In line with Green Chemistry and Circular Economy principles, an E-factor of 19.08 (about 9 with H_3_PO_4_, but with heat pre-treatment^13^), very close to that of the pharmaceutical industries and chemical industry^32^, was calculated taking into account that most of material involved can be recycled and valorized such as in the case of humins that represent a new platform for production of mesoporous carbons.

## Conclusion

Unprecedented in the literature, cigarette butts can be used as cellulose feedstock for producing Levulinic acid, one of the top value-added intermediates of chemical industry, by means of thermal hydrolysis (at 200 °C for 2 h) catalysed by phosphoric acid. The proposed protocol avoids the use of more aggressive H_2_SO_4_ and HCl, that are generally used for promoting this transformation from other cellulose sources (e.g. sludge paper), thus minimizing corrosion phenomena of plants. Further benefits that enable this method to be suitable for industrial applications are the following:

- reaction does not require neither pre-treatment of the starting material nor specific purification procedure of levulinic acid product;
- the possibility of recycling all the process components, ranging from humins by-products (by thermal valorisation), to the extracting solvent Ethyl acetate (by distillation), until to water phase residue (by Fenton COD abatement);
- the opportunity of obtaining, by simply modifying acid catalyst (e.g. using CH_3_COOH), another top value-added fine chemical such as 5-hydroxymethylfuraldehyde (HMF), thus widening the scope of the protocol.

Further advantages such as the huge amount of cigarette filters (megatonnes per years) that provide a no cost unlimited source of cellulose, suggest that this protocol marks a significant step forward compared to the current literature on this important issue.

## Materials and methods

### Materials

Ethyl acetate (> 99%) was purchased by Honeywell, Phosphoric acid (85%) and Levulinic acid were purchased from Sigma-Aldrich. All the reagents and solvents were used as received, without any further treatment. GC–MS analyses were run on a Shimadzu GLC 17-A instrument (Shimadzu, MI, Italy) using a SLB-5MS column (30 m × 0.25 mm id, film thickness 0.25 µm). Mass spectra were performed in EI mode (70 eV) and yields of LA were determined via GC–MS by means of a calibration curve (see supplemental information). NMR spectra were recorded on a Bruker 500 MHz spectrometer: ^1^H NMR (500 MHz) spectra were referenced to residual isotopic impurity of CDCl_3_ (7.25 ppm) and ^13^C-NMr (125 MHz) spectra were referenced to 77.00 ppm. Laser confocal scanning microscopy analyses were performed with an LSM-510 confocal microscope (Zeiss). ICP/MS analyses were carried out with a Thermo Fisher iCAP RQ (ICP-MS) instrument. COD analyses were performed with QuickCOD Labservice instrument. ATR-FTIR spectra *w*ere carried out on a Perkin-Elmer UATR-Two spectrophotometer instrument equipped with a single reflection diamond ATR crystal (refractive index of 2.4). Spectra were acquired with 32 scans in the range 4000—600 cm^−1^ by applying both the baseline and the ATR corrections.

### Levulinic acid synthesis

Weighed amounts of cigarette butts (250 mg ca. of “Rizla + ultra slim 5.7 mm”) were finely chopped in small pieces and suspended into 15 mL of aqueous H_3_PO_4_. Three different concentrations of H_3_PO_4_ were explored: 7.5% w/w, 15% w/w, and 20% w/w. Each suspension was charged into a 100 mL stainless steel autoclave and heated at temperatures in the range 160–260 °C for different times (1–6 h). After cooling, mixture was filtered and/or centrifugated to separate solid “Humins”, that were dried and weighed to give from 20 to 80% of yield (depending on the reaction conditions), while supernatant was extracted with ethyl acetate (2 × 20 mL). Combined organic phases were dried and the solvent removed in vacuo to give levulinic acid as crude oil.

Optimized procedure was then applied to washed and non-washed smoked cigarette butts recovered in Chemistry Department of Bari University, that were previously disinfected under UV rays and mechanically separated by the surrounding paper. A test was also carried out using unsmoked filter and 15 mL of aqueous CH_3_COOH 4 M in place of H_3_PO_4_ as catalyst. Used cigarette butts were washed in three cycles with 100 mL of water at 80 °C.

### Synthesis of levulinic acid on grams scale

To validate the protocol, reaction was repeated on grams scale. At this end, 5 g of unwashed smoked filter were treated, in autoclave, with 300 mL of aqueous H_3_PO_4_ 15% (w:w) for 2 h. Mixture was filtered and aqueous solution transferred into a separating funnel and extracted with Ethyl Acetate. The combined organic phases were distillated in vacuum to give 0.85 gr of Levulinic acid, while humins fraction was 1.4 g (corresponding to 28% w/w respect to the starting waste material).

Humins were characterized by ATR-FT /IR^28^ (see Supplemental Information), while Levulinic acid by ^1^H-NMR and ^13^C-NMR and GC/MS. All the spectra agreed with literature^33^ . Levulinic acid (**LA**): colorless liquid, bp 106–110 °C/6 mmHg; ^1^HNMR (CDCl_3_, 500 MHz): 2.72 (t, J = 6.5 Hz, 2H), 2.58 (t, J = 6.5 Hz, 2H), 2.16 (s, 3H); ^13^CNMR (CDCl3, 125 MHz): 206.6, 178.2, 37.7, 29.7, 27.8, GC/MS (70 eV) m/z (rel. intensity): 116.00 (M + , 2.74), 56.00 (28.82) 43.00 (100).

### Calculations and data analysis

Two different yields in Levulinic acid were calculated based on weight of filters and on theoretical amounts of LA. The first one, was calculated with the ratio Levulinic acid (g) obtained after the reaction/cigarette butts(g) × 100^14^.

The theoretical maximum yield^13,14^ of Levulinic acid is calculated on 250 mg of cigarette butts that contain 245 mg (98% ca.) of cellulose acetate (C.A.)^19^. Considering a 2:1 stoichiometric ratio of transformation (a dimeric C.A. unit leads to 2 molecules of Levulinic acid) and that C.A. dimeric unit molecular weight (MW_C.A._) is 492.428 mg/mmol., the C.A. millimoles can be calculated as follows:

$${\text{mmol}}_{{{\text{C}}.{\text{A}}}} \, = \,{\text{mg}}_{{{\text{C}}.{\text{A}}.}} /{\text{MW}}_{{{\text{C}}.{\text{A}}.}} \, = \,\left[ {{245} {\text{mg}}/{492}.{428} {\text{mg}}/{\text{mmol}}} \right]\, = \,0.{4975} {\text{mmol}}.$$

Cellulose acetate total conversion leads to 2 mol of L.A. (P.M_L.A_ = 116.11 mg/mmol) per cellulose acetate dimeric unit.

L.A. maximum millimoles and milligrams are obtained as follows:

$$\begin{gathered} {\text{mmol}}_{{{\text{L}}.{\text{A}}.}} \, = \,{\text{mmol}}_{{{\text{C}}.{\text{A}}.}} \, \times \,{2}\, = \,0.{995} {\text{mmol}}. \hfill \\ {\text{mg}}_{{{\text{L}}.{\text{A}}.}} \, = \,{\text{mm}}_{{{\text{L}}.{\text{A}}.}} \, \times \,{\text{ PM}}_{{{\text{L}}.{\text{A}}.}} \, = \,\left[ {0.{995} {\text{mmol}}\, \times \,{116}.{11} {\text{mg}}/{\text{ mmol}}} \right]\, = \,{115}.{54} {\text{mg}}. \hfill \\ \end{gathered}$$

Theoretical maximum yield (% W/W) can be calculated as follows:

$${\text{Rmax }}\left( \% \right)\, = \,\left( {{\text{mgL}}.{\text{A}}./{25}0 {\text{mg}}} \right)\, \times \,{1}00\, = \,\left( {{115}.{54} {\text{mg}}/{25}0 {\text{mg}}} \right)\, \times \,{1}00]\, = \,{46}.{2}\%$$

Theoretical yield was calculated as mm_L.Aex._/mm_L.Ath_  × 100.

The mm_L.Aex_ was obtained using the GC calibration curve in Supplemental Information.

### Determination of E-factor^4^

Mass of reactants: 2.2 g of H_3_PO_4_ (85%) in 15 mL of water (solvent (water) has been excluded from this calculation), cigarette filter 0.250 g; total amount of reactants 2.2 g + 0.250 g = 2.45 g.

$$\begin{gathered} {\text{Mass of product}}:0.0{43} {\text{g}}\,{\text{of}}\,{\text{ LA}}\, + \,0.0{79} {\text{g}}\,\,\,{\text{Humins}}\, = \,0.{122}. \hfill \\ {\text{Amount of waste}}: \, \left( {{2}.{45}{-}0.{122}} \right)\, = \,{2}.{38}. \hfill \\ {\text{E - factor}}\, = \,{\text{Amount of waste}}/{\text{Amount of product}}\, = \,{2}.{38}/0.{122}\, = \,{19}.0{8}. \hfill \\ \end{gathered}$$

## Supplementary Information

Supplementary Information.
