# Supplementary material for: Valorization of cigarette butts for synthesis of levulinic acid as top value-added chemicals
Source: Sci Rep. 2021 Aug 4;11:15775. doi: 10.1038/s41598-021-95361-4 (PMC8338950; doi:10.1038/s41598-021-95361-4)
Supplement: Supplementary file 1 — Supplementary Information. [file 41598_2021_95361_MOESM1_ESM.doc]

Supplemental Information

for

**Valorization of cigarette butts for synthesis of Levulinic Acid as top value-added chemicals**

Amelita G. Laurenza,^1^ Onofrio Losito,^2^ Michele Casiello,^2^ Caterina Fusco,^3^ Angelo Nacci,^2,3^ Vincenzo Pantone^1^ and Lucia D’Accolti^2,3*^

^1^ Ambra SRL Via Mitilini 11 Pomarico, Matera, 75016, Italy

^2^ Dipartimento di Chimica, Università degli Studi di Bari “A. Moro”, Via Orabona 4, 70126 Bari, Italy.

^3^ CNR – Istituto di Chimica dei Composti Organometallici (ICCOM), Bari section, via Orabona 4, 70126 Bari, Italy

*E-mail:* lucia.daccolti@uniba.it

**(10 pages, including this cover)**

###### Table of Contents

Calibration curve for determination of LA *p. S 2*

FTIR-ATR spectrum of Humins *p. S 3*

GC/MS analysis of reaction mixture with acid acetic as catalyst *p. S 4*

Cigarette butts characterization *p. S 5*

Laser confocal scanning microscopy (LCSM) analyses *p.* *S 5*

ICP/MS analyses *p. S 7*

COD analyses *p. S 8*

GC/MS analyses washing water of used cigarette butts……………………………………………………….….p. S 9

**Calibration curve for determination of LA**

To evaluate yields of levulinic acid (Tables 1-2), a calibration curve was prepared by means of 4 standard solutions of pure LA into ethyl acetate at different concentration. GC-MS areas were collected (50.0°C x 1min to 150.0 rate 25°C/min, to 170°C rate 20°C/min, to 250 rate 80°C x 20 min) for each solution by means of three replicates as reported in Fig. S1.

| Standard soln. | C (% w/w) | Areas | SD |
| --- | --- | --- | --- |
| STD #1 | 0.2613 | 3834648 | 180601 |
|  |  | 3583892 |  |
|  |  | 3934418 |  |
| STD #2 | 0.5195 | 7493909 | 250024 |
|  |  | 7027383 |  |
|  |  | 7104758 |  |
| STD #3 | 0.7525 | 11703311 | 173357 |
|  |  | 11589283 |  |
|  |  | 11362738 |  |
| STD #4 | 0.9840 | 15738782 | 225755 |
|  |  | 15552728 |  |
|  |  | 15289478 |  |

*Figure S1.* Calibration curve for evaluating yields of Levulinic Acid.

**FTIR-ATR spectrum of Humins**

Humins by-products were identified by ATR-FT /IR by comparison with the literature (Fig. S2, see ref. 25).


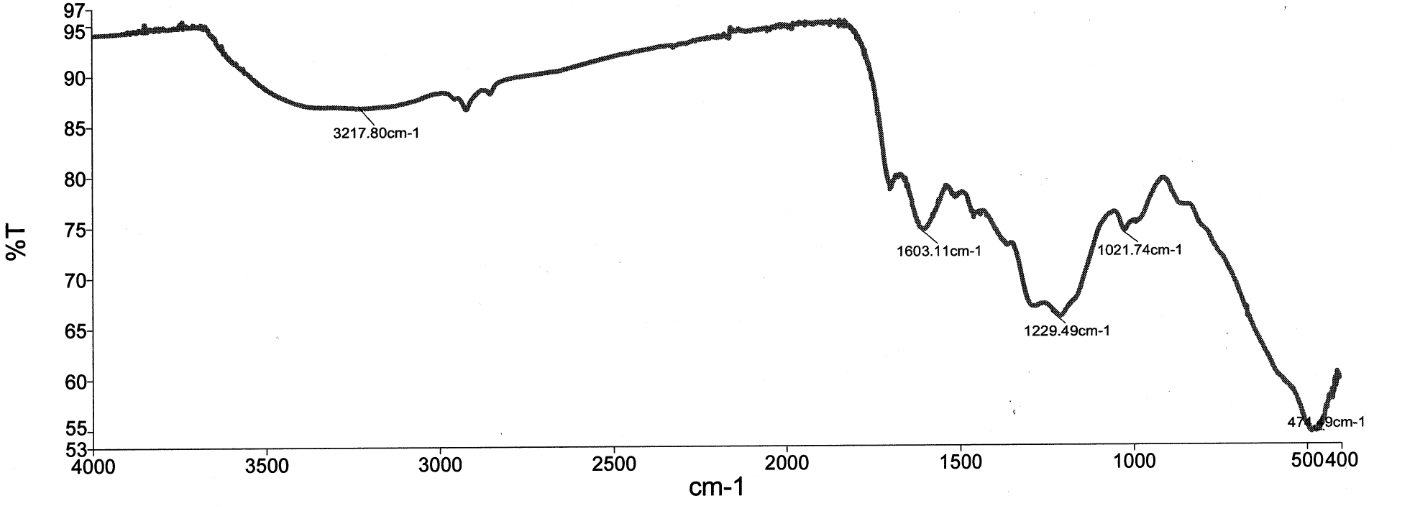


*Figure S2*. FTIR-ATR of Humins from smoked filter.

**GC/MS analysis of reaction mixture with acid acetic as catalyst**

GC/MS analysis of reaction mixture (Fig. S3) revealed the presence of 5-hydroxymethylfurfural (5-HMF) as the sole reaction product when acetic acid was used as the catalyst (Table 1, entry 15). Identification of 5-HMF was carried out with the aid of NIST database (Fig. S4).

*Figure S3.* GC/MS analysis of reaction mixture on unsmoked CBs catalysed by CH_3_COOH (50.0°C x 1min to 150.0 rate 25°C/min, to 170°C rate 20°C/min, to 250 rate 80°C x 20 min)


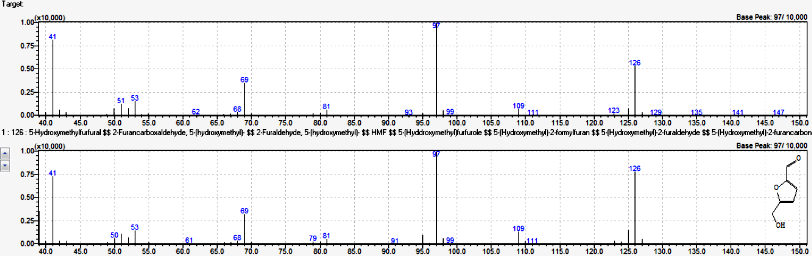


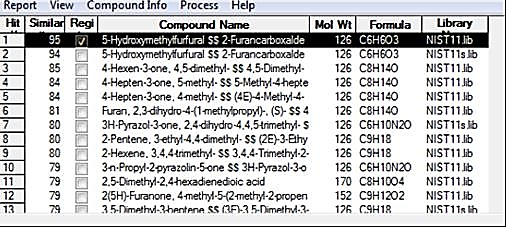


*Figure S4.* Identification of 5-HMF with NIST database.

**Cigarette butts characterization**

Starting cellulosic material was characterized by LCSM technique for determining fibres morphology, and by ICP/MS and COD analyses for evaluating amounts of contaminants.

**Laser confocal scanning microscopy (LCSM) analyses**

LCSM image of unsmoked CBs revealed a structure of ordered cellulose acetate fibres with a helical arrangement possessing thickness of 26.3 μm and 31.6 μm and a large space between them (Fig. S5). In contrast, after smoking, fibers seemed to agglomerate into more compact and disordered structures with a decreased interspace (Fig. S6). Samples were illuminated with a 488nm laser, and the reflected light was collected with a PMT. Fluorescence information was simultaneously collected between 425-470nm using an excitation wavelength of 405nm.


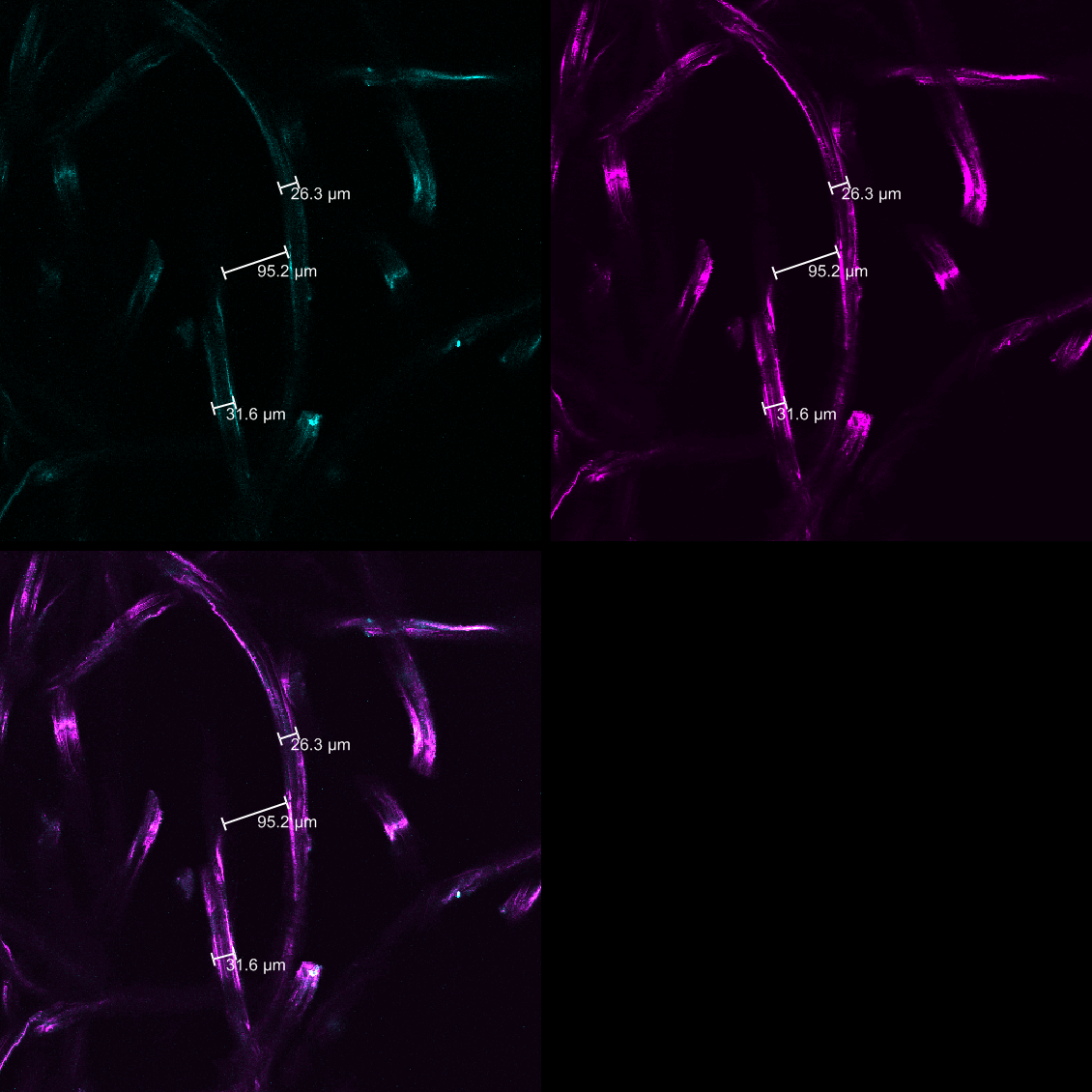


*Figure S5.* Image of unsmoked cigarette butt by confocal laser scanning microscopy.


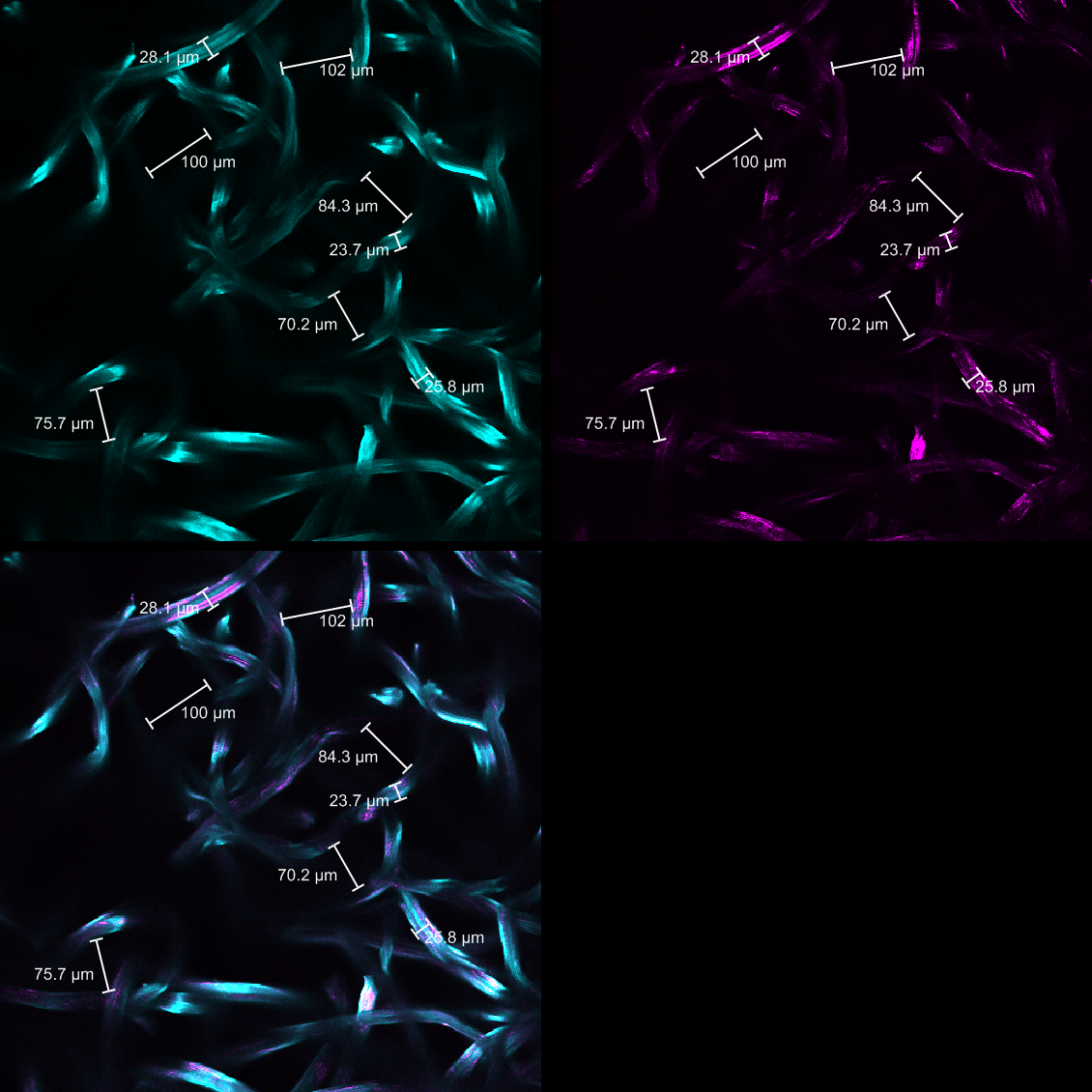


*Figure S6.* Image of smoked cigarette butt by confocal laser scanning microscopy.

This change of structure could be due to the cleavage of acetate functionalities of cellulosic chains after smoking enabling the formation of interchains H-bonds (or ionic bonds).

**ICP/MS analyses**

ICP/MS analyses were executed on both unsmoked and smoked CBs, and for the latter before and after washing. Results are listed in Table S1. As expected, filters retained large quantities of many metals during smoking. Only a little part of them is removed by the preliminary washing. Most of these metals remain into the water phase residue after extraction of LA with ethyl acetate and can be removed upon treatment with Ca(OH)_2_ during phosphate precipitation (see Fig. 5).

Table S1. ICP/MS analyses of cigarette butts.

| Metal^a^ | Unsmoked Butts  (ppm) | Smoked Butts  (ppm) | Smoked Butts  after washing (ppm) |
| --- | --- | --- | --- |
| As | 37 | 52 | 40.4 |
| Cd | 121 | 48 | 42 |
| Al | 11849 | 22336 | 19937 |
| Pb | 218 | 179 | 173 |
| Ni | 1080 | 1228 | 1204 |
| Ba | 449 | 447 | 325 |
| B | 2586 | 4117 | 3633 |
| Ca | 12480 | 19937 | 18313 |
| Fe | 13712 | 68881 | 36066 |
| Mn | 299 | 897 | 586 |
| V | 472 | 484 | 412 |
| Hg | 204 | 220 | 216 |
| Cu | 420 | 1375 | 625 |
| Zn | 10293 | 27419 | 9434 |
| Sn | 10553 | 11784 | 3984 |
| Se | 303 | 894 | 492 |
| Cr | 599 | 1884 | 1368 |
| Co | 38 | 109 | 104 |
| Mo | 80 | 148 | 137 |

^a^ Determined on 50 mg of filter in 20 mL in water.

**COD analyses**

Other contaminants were monitored by measuring chemical oxygen demand (COD) and performing GC-MS analyses of washing water. The former technique revealed an average COD value of 1167 ppm (Fig. S7).


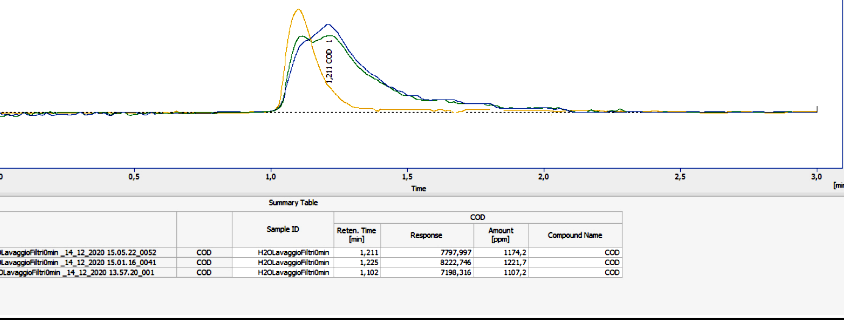


*Figure S7*. COD analyses of washing water of used cigarette butts.

GC-MS analyses of washing water showed triacetin as major contaminant (Fig. S8), an expected result given that this compound is a common additive (humectant) of cigarette filters. Trace amounts of hydrolyzed triacetins and polyphenols were also detected (Fig. S9).


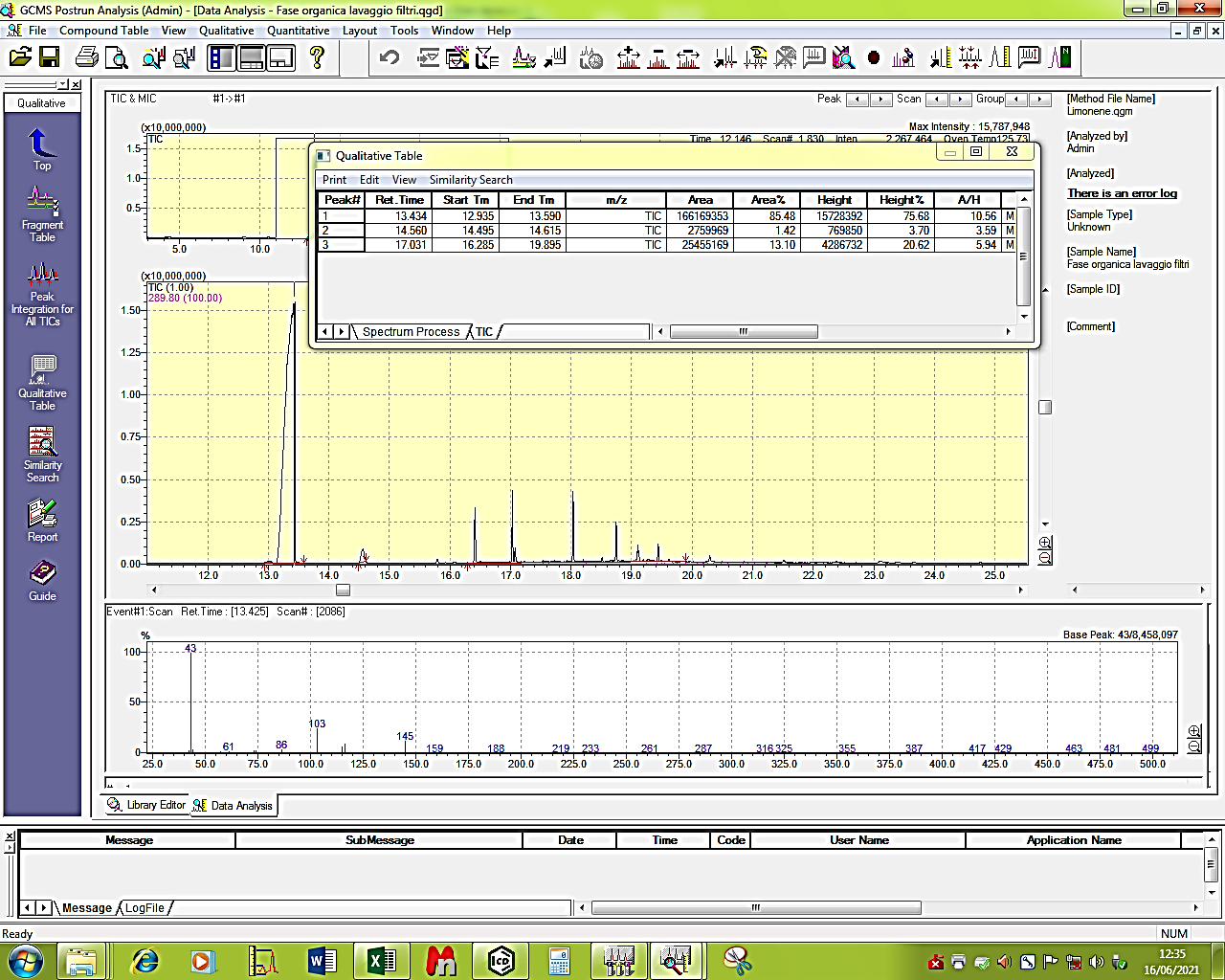


*Figure S8*. GC/MS analysis of washing water of used cigarette butts.


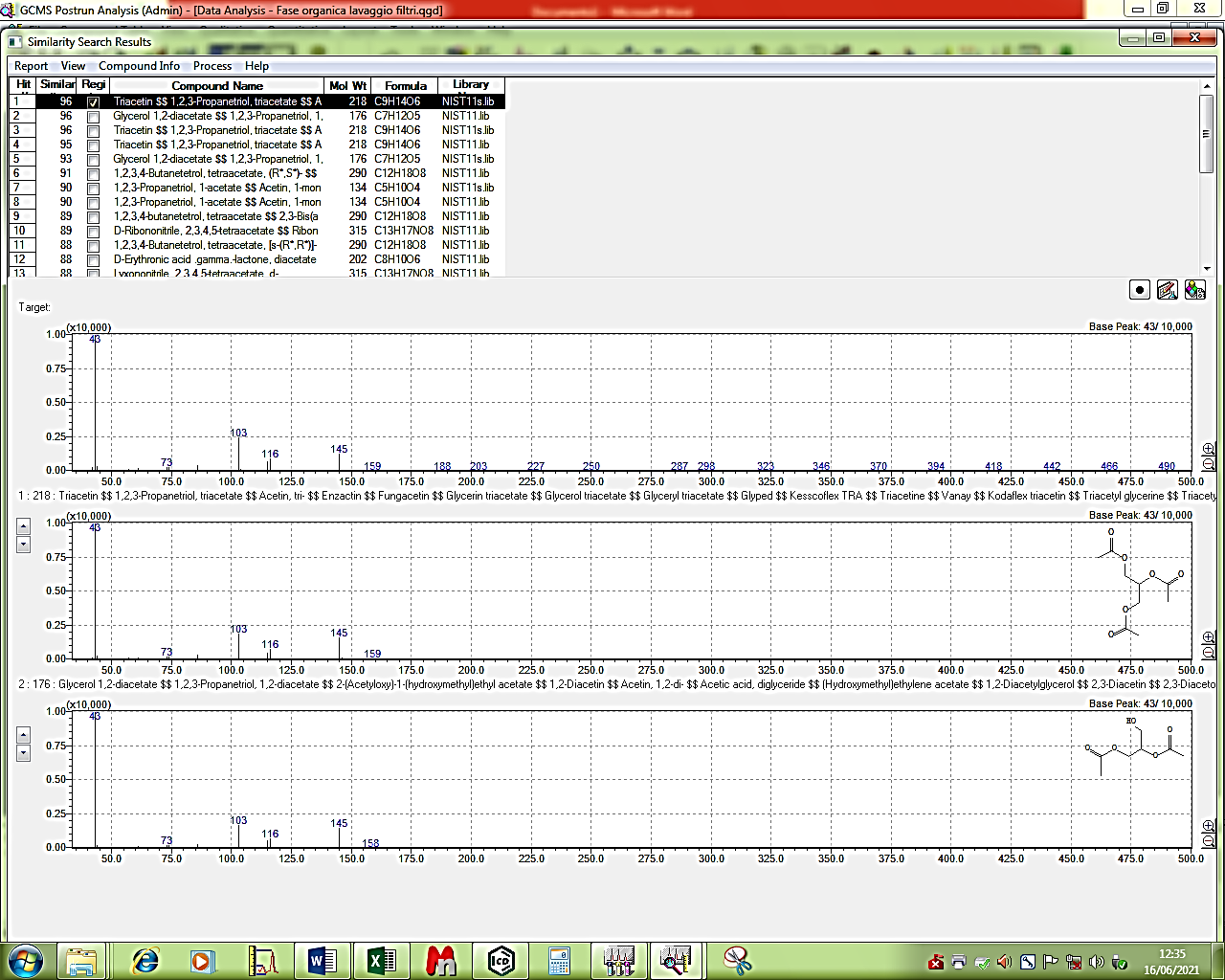


*Figure S9.* MS spectrum of major component of washing water of smoked cigarette butts.
